# Treatment outcomes among HIV-1 and HIV-2 infected children initiating antiretroviral therapy in a concentrated low prevalence setting in West Africa

**DOI:** 10.1186/1471-2431-12-95

**Published:** 2012-07-08

**Authors:** Uduak Okomo, Toyin Togun, Francis Oko, Kevin Peterson, John Townend, Ingrid Peterson, Assan Jaye

**Affiliations:** 1 Medical Research Council (UK) Laboratories, Atlantic Road, Fajara, 273, Banjul, The Gambia; 2 Institute of Tropical Medicine, Nationalestraat 155, Antwerp, 2000, Belgium; 3 International Center for AIDS Care and Treatment Program (ICAP)-Swaziland, Mailman School of Public Health Columbia University, New York, USA; 4 WAPHIR Network, Universite Cheikh Anta Diop Laboratoire de Bacteriologie Virologie, Hopital A le Dantec, 30 Avenue Pasteur, Dakar, Senegal

## Abstract

**Background:**

There is little data on responses to combination antiretroviral therapy (cART) among HIV-infected children in the West African region. We describe treatment outcomes among HIV-1 and HIV-2 infected children initiating cART in a research clinic in The Gambia, West Africa.

**Methods:**

All treatment naive HIV-infected children who initiated cART according to the WHO ART guidelines for children between October 2004 and December 2009 were included in the analysis. Kaplan-Meir estimates and sign-rank test were used to investigate the responses to treatment.

**Results:**

65 HIV-1 and five HIV-2 infected children aged < 15 years were initiated on cART over this time period. HIV-1 infected children were treated with a combination of Zidovudine or Stavudine + Lamivudine + Nevirapine or Efavirenz while children with HIV-2 were treated with Zidovudine + Lamivudine + ritonavir-boosted Lopinavir. HIV-1 infected children were followed-up for a median (IQR) duration of 20.1 months (6.9 – 34.3), with their median (IQR) age at treatment initiation, CD4% and plasma viral load at baseline found to be 4.9 years (2.1 – 9.1), 13.0% (7.0 – 16.0) and 5.4 log_10_ copies/ml (4.4 – 6.0) respectively. The median age at treatment initiation of the five HIV-2 infected children was 12 years (range: 4.6 – 14.0) while their median baseline CD4^+^ T cell count and HIV-2 viral load were 140 cells/mm^3^ (Range: 40 – 570 cells/mm^3^) and 4.5 log_10_copies/mL (Range: 3.1 - 4.9 log_10_copies/mL) respectively.

Among HIV-1 infected children <5 years of age at ART initiation, the median (IQR) increases in CD4% from baseline to 12, 24 and 36 months were 14% (8 – 19; *P* = 0.0004), 21% (15 – 22; *P* = 0.005) and 15% (15 – 25; *P* = 0.0422) respectively, while the median (IQR) increase in absolute CD4 T cell count from baseline to 12, 24 and 36 months for those ≥5 years at ART initiation were 470 cells/mm^3^ (270 – 650; P = 0.0005), 230 cells/mm^3^ (30 – 610; P = 0.0196) and 615 cells/mm^3^ (250 – 1060; P = 0.0180) respectively. The proportions of children achieving undetectable HIV-1 viral load at 6-, 12-, 24- and 36 months of treatment were 24/38 (63.2%), 20/36 (55.6%), 8/22 (36.4%) and 7/12 (58.3%) respectively. The probability of survival among HIV-1 infected children after 12 months on ART was 89.9% (95% CI 78.8 – 95.3). CD4 T cell recovery was sub-optimal in all the HIV-2 infected children and none achieved virologic suppression. Two of the HIV-2 infected children died within 6 months of starting treatment while the remaining three were lost to follow-up.

**Conclusions:**

The beneficial effects of cART among HIV-1 infected children in our setting are sustained in the first 24 months of treatment with a significant improvement in survival experience up to 36 months; however the outcome was poor in the few HIV-2 infected children initiated on cART.

## Background

Initiatives to increase access to antiretroviral therapy (ART) for paediatric populations in resource-limited settings, particularly the integration of HIV treatment and care for children into existing adult ART centres as well as maternal newborn and child health services, have resulted in a significant increase in the numbers of children starting treatment over the last couple of years [1]. In Sub-Saharan Africa where the global paediatric HIV burden is situated there are however wide sub-regional differences in paediatric ART coverage with greater access seen in high-prevalence countries in Eastern and Southern Africa compared with Western and Central Africa [1].

Gambia has a concentrated but low-level mixed epidemic with estimated prevalence of HIV-1 and HIV-2 infection of 1.6% and 0.4% respectively from the 2008 national sentinel surveillance data [1,2]. Confined mostly to West Africa, HIV-2 is noted to be less pathogenic than HIV-1 with prolonged asymptomatic phase [3,4] however, disease progression to HIV-2 associated-AIDS does occur and is indistinguishable from that of HIV-1 [5,6]. In the absence of preventive interventions, perinatal transmission rates of HIV-2 range from 0 – 4% [7-10]. It is estimated that there are currently fewer than 1000 children under 15 years of age known to be living with HIV in The Gambia of whom approximately one-third are receiving life-saving ART [11]; however, sub-optimal coverage of prevention of mother-to-child-transmission (PMTCT) services nation-wide, poor follow-up of HIV-exposed infants and children and the general inability to perform diagnostic virologic tests on infants < 18 months of age suggest that HIV-infected children are underestimated.

Data on treatment outcomes from paediatric ART programmes in the West African sub-region is limited to countries with a high burden of paediatric HIV, and describe only HIV-1 infected children [12-15]. Very few studies have described survival of ART-naïve HIV-2-infected children [16,17], and in contrast to HIV-1, little is known about the best approach to the clinical treatment and care of children infected with HIV-2 using antiretroviral drugs developed against HIV-1[18].

We report the experiences of ART-naïve HIV-1 and HIV-2 infected Gambian children starting ART at a major paediatric HIV referral clinic at the Medical Research Council (MRC) Unit in Fajara, The Gambia – West Africa.

## Methods

### Study setting and participants

The MRC Fajara HIV clinical cohort was established in 1986 with approval from the Joint Gambia Government/ MRC Ethics Committee and enrolment of children into the cohort started in 1993, based on written informed consent by the parent or legal guardian. The cohort initially consisted of children whose HIV-infected parents were enrolled in a natural history cohort, and later children identified as HIV-infected and clinically in need of treatment referred from other health-care facilities. The Fajara clinic has been the only centre in the country performing virological tests for the diagnosis of HIV infection in children <18 months of age and became the major paediatric HIV referral clinic in The Gambia. Provision of ART in The Gambia began in October 2004 through the support of the Global Fund to fight AIDS, Tuberculosis and Malaria. Recruitment of children into the programme ended in January 2010 as part of the transition process transfer patient care from the MRC to the Gambian national health care system.

**Figure 1:**
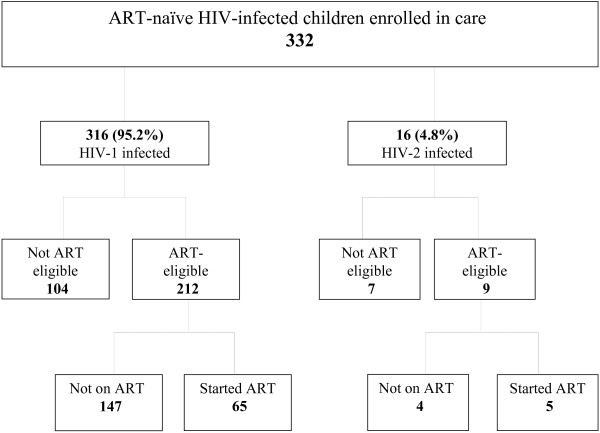
Flowchart of ART-naïve HIV-infected children < 15 years of age enrolled in the MRC Fajara paediatric HIV programme from June 2004 - September 2009.

Between June 2004 (when sensitization of patients in preparation for the introduction of ART commenced) and December 2009, a total of 332 ART-naïve HIV-infected children were enrolled in the paediatric cohort of whom 316 (95.2%) were HIV-1 and 16 (4.8%) HIV-2 positive (Figure 1). At enrolment, data on socio-demographic characteristics were collected and a baseline assessment conducted to determine clinical stage and screen for opportunistic/concomitant infections; CD4 T-cell profile, complete blood count and serum biochemical indices were also measured. All clinical services and treatments (including ART) were provided free of charge and the patients also received reimbursement of their transport costs to and from the clinic at every clinic/ counselling visit whether on ART or not. Under the orphans and vulnerable children (OVC) programme, nutritional support was provided, including infant milk formula for children up to 18 months of age whose mothers opted not to breast-feed, as well as energy-rich complementary feeds. School fees were also paid for all children enrolled in school up till the age of 18 years.

### Study procedures

Eligibility for initiating ART was based on the standard WHO guidelines that were in place at the time of enrolment [19,20]. In addition, all children were required to have at least one identifiable caregiver who would take responsibility for the administration of the child’s medications. Caregivers underwent a minimum of four pre-ART counselling sessions over a period of three to six weeks. ART was initiated only after approval by a national ART eligibility committee which reviewed the clinical and social data of all children whose caregivers had completed the pre-treatment counselling. At initiation of therapy, a baseline physical assessment was conducted and a blood sample drawn for determination of baseline CD4^+^ T cell profile, HIV RNA plasma viral load, complete blood count and serum chemistry.

### Antiretroviral therapy

ART was initiated in accordance with The Gambian Department of State for Health guidelines [21]. The first-line regimen for HIV-1 infected children consisted of zidovudine (AZT) and lamivudine (3TC) in combination with nevirapine (NVP). AZT was replaced by Stavudine (d4T) for children with a haemoglobin < 8 g/dl while NVP was replaced by Efavirenz (EFV) for children over 3 years of age (or >10Kg) who were receiving concurrent treatment for tuberculosis. The first-line regimen for HIV-2 infected children consisted of a combination of AZT, 3TC and ritonavir-boosted lopinavir (LPV/r). Drugs were administered as syrups, single-drug formulations, alone or in combination with adult, generic fixed-dose combination tablets cut in half or quartered. All children received daily cotrimoxazole prophylaxis in accordance with WHO guidelines [22,23].

### Follow-up

Following ART initiation children were followed up at 2 weeks, 4 weeks, and then at monthly intervals for the first 3 months and quarterly thereafter. At each visit, patients were seen by a physician. Haematologic tests and biochemical tests for liver function were performed at the week 4 and quarterly follow-up visits. CD4^+^ T cell profile, HIV RNA plasma viral load were measured at 3- and 6-months and thereafter every 6 months or as clinically indicated. Data on drug dispensing was collected in the pharmacy registry database. Adherence was assessed at each visit by pill counts (for children receiving tablets) and/or self report of the care-giver or child. Patients with observed adherence problems were offered further counselling. Immunologic and virologic responses to ART were assessed by changes in the CD4^+^ T cell parameters and plasma viral load levels respectively from the pre-therapy levels. An undetectable viral load was defined as being <100 copies/mL. Patients who did not come to the clinic for at least 90 days beyond their last drug-refill visit were considered lost to follow up and where prior consent obtained, were visited at home by trained field workers to ascertain survival status or change of address.

### Laboratory methods

#### HIV diagnosis

Sequential assays were carried out for serological diagnosis of HIV in children ≥18 months of age. Murex HIV antibody test (ELISA) were carried out first on all serum samples; a positive sample was presumed to contain antibodies to HIV 1 or HIV-2. This was followed by Hexagon HIV test for qualitative detection of IgG, IgA and IgM antibodies to HIV-1 and HIV-2, which differentiates between HIV-1 and HIV-2 seropositivity. A coloured band at line 2 and a coloured band at the control line indicate HIV-2 seropositivity. In children <18 months of age, HIV infection was diagnosed by two polymerase chain reaction (PCR) tests. The MRC Fajara HIV clinic is the only centre in The Gambia performing virological tests for the diagnosis of HIV infection in children <18 months of age.

### HIV RNA viral load

Viral load testing was done using a quantitative polymerase chain reaction method with 100 – 1,000,000 copies/ml as the limit of detection. This method of viral load evaluation was developed in-house for use in the MRC Laboratories, The Gambia [24]. Genotypic resistance testing was not available due to cost.

### CD4 count measurement

Percentages and absolute counts of CD4 T lymphocytes were determined on a FACS Calibur (Becton Dickinson, US) using BD MultiTest reagents and MultiSet software (BD Immunocytometry Systems).

### Statistical analysis

All treatment-naive children who initiated ART at the MRC HIV clinic between 1 October 2004 and 31 December 2009 were included in this analysis; children transferred into the cohort having been on ART elsewhere were excluded. For the purpose of this analysis, children who initiated treatment before 2006 were retrospectively re-staged at the time of starting ART according to the 4-stage WHO paediatric HIV clinical classification. Observation time for each child started at the date of commencement of ART and ended either on the 31^st^ of December 2009 or at the date of death or the date the child was last known to be alive, whichever came first.

The main outcomes for this study were changes in immunologic and virologic parameters measured after initiating ART. Baseline parameters were taken as the nearest measurement within 3 months prior to initiation of ART; whereas the subsequent 6-monthly monitoring tests parameters were the nearest measurement within a ± 3-month window of the respective time point after initiation of ART. Differences between baseline and each 6-monthly follow-up CD4 measurement were determined using the Wilcoxon signed test. Kaplan-Meier estimates were used to determine the time to achievement of an immunological response to ART and probability of survival. The time to achieve an immunological response was defined as the interval between the baseline CD4% and an increase in the CD4% of 5-, 10-, and 15-percentage points. An undetectable viral load was defined as a viral load of < 100 copies/ml. The proportion of children achieving undetectable viral load after 3-, 6-, 12-, 18-, 24-, 30- and 36 months of treatment respectively was estimated for all children with available measurements at each specified time-point. Virological failure was defined as having HIV RNA viral load >5000 copies/mL after 24 weeks (6 months) of therapy. Differences in baseline viral load and CD4^+^ T cell profiles between children who died and those who survived were assessed using Wilcoxon rank sum tests. All statistical analyses were performed with STATA Version11 (Stata Corp., College Station, TX, USA) and statistical significance defined as p<0.05 (two-sided).

## Results

Between October 1, 2004 and December 31, 2009, 70 treatment-naïve HIV- infected children less than 15 years of age were initiated on ART in the MRC paediatric HIV cohort. Sixty-five (92.9%) children were HIV-1 infected and 5 (7.1%) HIV-2 infected. The children were followed up for a median duration of 18.2 months (IQR: 5.7 – 34.3 months) during which time a total of eight children (11.4%) died and 12 (17.1%) were lost to follow-up.

### Baseline characteristics of HIV-infected children on ART

#### Characteristics of HIV-1-infected children

The 65 HIV-1 infected children had a median duration of follow-up of 20.1 months (IQR: 6.9 – 34.3 months) with 50 (76.9%) of the children having at least 6 months follow-up after the start of ART. Thirty-one children (47.7%) were female. The median age at start of treatment was 4.9 years (IQR: 2.1 - 9.1 years); 15 children (23.1%) were less than 2 years of age. Twenty-seven (41.5%) children had advanced (stage 3) or severe (stage 4) clinical disease, 18 of whom were also severely immunosuppressed (baseline CD4% <15%) and 9 of whom had pulmonary tuberculosis. Baseline characteristics of the HIV-1 infected children are presented in Table 1.

**Table 1:** Baseline characteristics of HIV-1 infected children starting ART in the MRC Paediatric HIV Cohort, The Gambia

**Characteristics**	**HIV-1 (n = 65)**
Male [No (%)]	34 (52.3%)
Female [No (%)]	31 (47.7%)
Age, years [median (IQR:)]	4.9 (2.2 – 9.1)
Age distribution [No. (%)]	
< 2 years	15 (23.1%)
2 – 4 years	19 (29.2%)
≥ 5 years	31 (47.7%)
Parent status [No. (%)]	
Both parents alive	34 (53.3%)
Only mother living	6 (9.2%)
Only father living	16 (24.6%)
Both parents dead	5 (7.7%)
Unknown	4 (6.2%)
WHO Paediatric Clinical stage [No. (%)]	
Stage 1	26 (40.6%)
Stage 2	12 (18.5%)
Stage 3	19 (29.2%)
Stage 4	8 (12.3%)
Immunologic category (CD4^+%^) [No. (%)]	
>25%	1 (1.6%)
15% – 25%	25 (39.7%)
<15%	37 (58.7%)
Baseline CD4^+^% [median (IQR); n = 63 ^a^]	13 (7 – 16)
< 2 years (n = 14)	13 (6 – 16)
2 – 4 years (n = 18)	14.5 (11 – 18)
≥ 5 years (n = 31)	10 (7 – 15)
Baseline CD4^+^ count [median (IQR); n = 62 ^b^]	390 (210 – 730)
< 2 years (n = 13)	510 (300 – 1100)
2 – 4 years (n = 18)	615 (280 – 1020)
≥ 5 years (n = 31)	285 (100 – 520)
Baseline Viral load, log_10_copies/mL [median (IQR); n = 55^c^]	5.4 (4.5 – 6.0)

^a^2 missing values; ^b^3 missing values; ^c^10 missing values.

Both parents of seven (10.8%) children were known to be HIV-positive; 41 (63.1%) children had at least one parent known to be HIV-positive; and the HIV status of both parents were negative or unknown in 3 (4.6%) and 14 (21.5%) of the children respectively. Five (7.7%) children had lost both parents, 34 (52.3%) had both parents alive while 22 had lost at least one parent.

Fifty-four (83.1%) children were commenced on AZT + 3TC + NVP; six (9.2%) on AZT + 3TC + EFV and five (7.7%) on d4T + 3TC + NVP. Twelve (18.5%) of the children had a substitution of at least one antiretroviral drug while 4 (6.2%) among the 12 subsequently had a second drug substitution during follow-up. The main reasons for drug substitution were severe anaemia and/or treatment for tuberculosis.

### Characteristics of HIV-2-infected children

The median duration of follow-up among the HIV-2-infected children was 3.8 months (Range: 1.8 – 37.5 months). Three of the children were male and 2 were female. The baseline clinical and laboratory characteristics of the five HIV-2 infected children are presented in Table 2. Their median age at the start of treatment was 12 years (Range: 4.6 – 14.0 years); four (80%) of the children had advanced or severe clinical HIV disease at the start of treatment. The median baseline CD4^+^ T cell count and baseline HIV-2 viral load were 140 cells/mm^3^ (Range: 40 – 570 cells/mm^3^) and 4.5 log_10_copies/mL (Range: 3.1 - 4.9 log_10_copies/mL) respectively.

**Table 2:** Demographic, laboratory and clinical summary of HIV-2 infected children on antiretroviral therapy

**Patient**	**Sex**	**Age at diagnosis (years)**	**Parents status**	**Age at start of ART (years)**	**Baseline CD4**^**+**^**count (%CD4)**	**Baseline Viral load (log**_**10**_**copies/ml)**	**Remarks**
1	F	11	Both parents alive; HIV status unknown	12	40 (1%)	Nil	WHO stage 4 at start of ART Clinical follow-up for 2 months No follow-up laboratory data; Died
2	M	8	Mother dead (HIV-2 positive), Father alive (HIV-negative)	14	280 (27%)	4.5	WHO stage 4 at start of ART Clinical follow-up for 38 months Sub-optimal CD4 recovery; >0.5 log_10_ increase in viral load at 12 weeks; did not achieve virologic suppression throughout follow-up; LTFU
4	F	2	Both parents HIV-2 positive and alive (both on ART)	4	570 (13%)	4.6	WHO stage 3 at start of ART Clinical follow-up for 10 months No CD4 cell gain; ART discontinued at 29 weeks due to poor adherence; No follow-up viral load data; LTFU
5	M	6	Mother dead, father unknown; both parents HIV status unknown	6	140 (4%)	3.1	WHO stage 3 at start of ART Clinical follow-up for 4 months No follow-up laboratory data; Died
6	M	12	Mother dead, father unknown; both parents HIV status unknown	12	110 (8%)	4.9	WHO stage 2 at start of ART Clinical follow-up for 3 months Achieved an increase in CD4 count of 50 cells/mm^3^ but a 1.0 log_10_ increase in viral load at 12 weeks; LTFU

NOTE. LTFU = Lost to follow-up; EPTB = Extra pulmonary tuberculosis; PTB = Pulmonary tuberculosis.

### Response to treatment among HIV-1-infected children

#### Immunologic response to treatment

Overall, the median (IQR) CD4% increased significantly from 13% (7 – 16) at baseline to 23.3% (18.0 – 28.5; p < 0.001), 27% (20 – 34; p < 0.001), and 27.5% (16 – 36; p < 0.001) at 6-, 12- and 18 months respectively (Table 3).

**Table 3:** Immunologic response to antiretroviral therapy among HIV-1-infected children

**Increase in CD4% from baseline following initiation of ART in all children (n = 65)**				
**Duration of follow-up (months)**	**Number of children**^**a**^	**Median CD4% (IQR)**	**Median CD4% increase from baseline (IQR)**	***P***^***b***^
6	42	23.3% (18.0 – 28.5)	11% (4 – 15)	< 0.001
12	37	27% (20 – 34)	15% (7 – 20)	< 0.001
18	30	27.5% (16 – 36)	17% (6 – 22)	< 0.001
24	25	29% (21 – 38)	18.5% (5 – 22)	0.0001
30	19	32% (19 – 38)	15% (9 – 24)	0.0001
36	12	30.3% (22.5 – 39.5)	18.5% (10.8 – 24.5)	0.002

^a^ Number of children tested at corresponding length of follow-up. Attrition in numbers is attributable to lack of data, loss to follow-up or death.

^b^ Wilcoxon signed ranks test.

**Figure 2:**
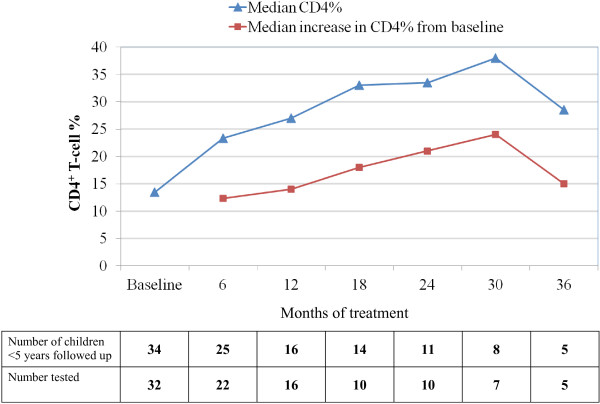
**CD4% changes of HIV-1 infected children in the MRC Fajara cohort in response to ART.** Median CD4% and median increase in CD4% from baseline among HIV-1 infected children <5 years of age at ART start.

Among those aged less than 5 years at initiation of ART, the median (IQR) CD4% increase from baseline to 6-, 12 and 18 months of therapy was 12.3% (8 – 14; P < 0.001), 14% (8 – 19; P = 0.0004) and 18% (13 – 22; p = 0.0048) respectively. (Figure 2)

**Figure 3:**
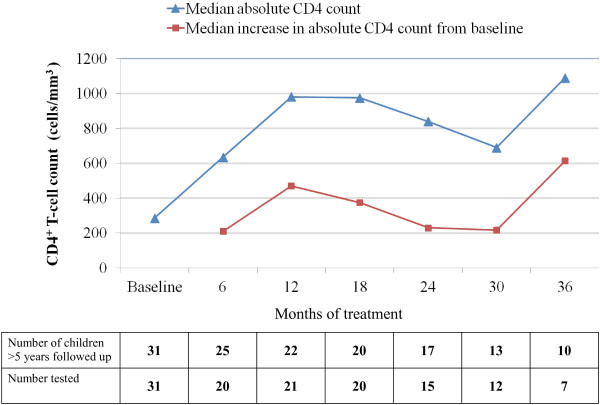
**Absolute CD4 T-cell count changes of HIV-1 infected children in the MRC Fajara cohort in response to ART.** Median absolute CD4 T-cell count and median changes in CD4 T-cell from baseline among HIV-1 infected children ≥5 years of age at ART start.

CD4^+^ T cell count were used to assess rate of immunological recovery in children ≥ 5 years of age at the start of ART. The median (IQR) increase in absolute CD4^+^ T cell count from baseline to 6-, 12- and 18 months of therapy was 210 cells/mm^3^ (67.5 – 380; P = 0.0009), 470 cells/mm^3^ (270 – 650; p = 0.0005) and 375 cells/mm^3^ (−62.5 – 835; p = 0.0085). (Figure 3)

**Figure 4:**
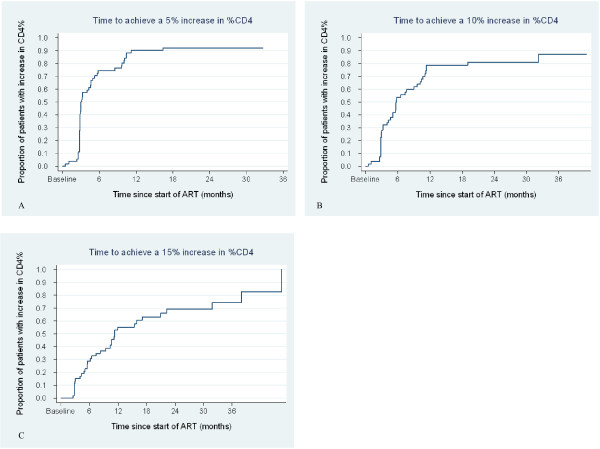
**Kaplan Meier plots for increase in CD4% from baseline among HIV-1 infected children.** Kaplan Meier survival curves of increase in CD4% from baseline with time with plots A, B and C showing time to (A) 5%, (B) 10% and (C) 15% increase in CD4% from baseline.

The Kaplan-Meir curves in figures 4A – C show the evolution of the CD4% of the all HIV-1-infected children on ART during the follow-up period. The median times to achieve 5%, 10% and 15% increases in the CD4% were 2.9 months (IQR: 2.8 – 5.8); 5.6 months (IQR: 2.9 – 11.1) and 10.3 months (5.5 – 17.1) respectively.

### Virologic response to treatment

At Six months of treatment, 24 (63.6%) of the 38 children with available viral load measurements had achieved complete virologic suppression (i.e. a viral load <100 copies/mL); 7 (18.4%) children had virologic failure (i.e. a viral load > 5000 copies/mL), while 7 (18.4%) children had incomplete virologic suppression (i.e. 5000 > viral load >100 copies/mL). The suppression of detectable viral load was achieved in 55.6%, 36.4% and 58.3% of children at 12, 24 and 36 months of follow-up respectively (Table 4).

**Table 4:** Virologic response to antiretroviral therapy among HIV-1-infected children

**Duration of follow-up, months**	**Children with viral load measurement**^**a**^**(%)**	**Children with HIV-1 viral load <100 copies/ml (%)**^**b**^
3	39/54 (72.2%)	21 (53.9%)
6	38/50 (76%)	24 (63.2%)
12	36/38 (94.7%)	20 (55.6%)
18	27/34 (79.4%)	14 (51.9%)
24	22/28 (78.6%)	8 (36.4%)
30	18/21 (85.7%)	9 (50%)
36	12/16 (75%)	7 (58.3%)

^a^ Denominators represent the number of children with the corresponding length of follow-up.

^b^ Percentage of those with a viral load measurement at each time point.

During the period of follow-up, only eight children were switched to second-line therapy as a result of treatment (virologic) failure); four (50%) of these children were less than 5 years of age when they started first-line therapy. The median time to initiation of second-line therapy was 23.5 months (IQR: 15.8 – 30.7). Six of these children eventually achieved complete virologic suppression whilst the remaining two achieved viral loads <5000 copies/mL.

### Clinical outcomes

**Figure 5:**
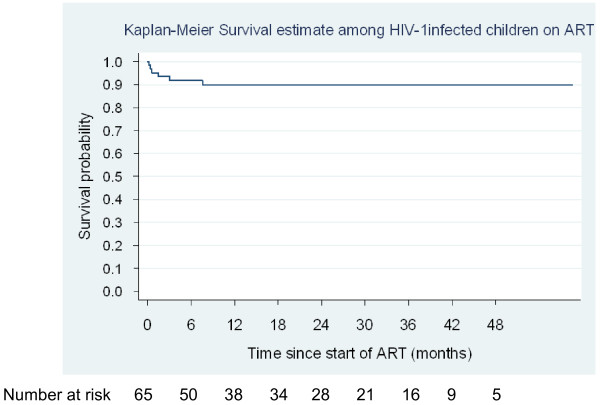
Kaplan Meier plot showing survival probability among HIV-1 infected children on ART in the MRC Fajara Paediatric cohort, The Gambia.

One child was transferred to another ART centre for continued care and 8 (12.3%) were lost to follow-up of whom one was withdrawn from the programme to seek alternative (herbal) treatment. Six (9.2%) HIV-1-infected children died after initiating ART; the median time to death was 31.5 days (range: 6 – 231 days). Five (83.3%) of the deaths occurred within 6 months of starting treatment; four of which took place in within the first 90 days. All the children but one were < 2 years of age at the time of death however, all had WHO clinical stage 3 or 4 disease. Children who died had significantly lower CD4% at baseline than those who survived (3.5% vs. 13% respectively; P = 0.004), while baseline viral loads did not differ (5.7 vs. 5.3 log_10_copies/mL respectively; P = 0.647). The probability of survival on ART among the HIV-1-infected children after 12 months was 89.9% (95% CI 78.8 – 95.3%) (Figure 5).

#### Response to treatment among HIV-2-infected children

Two of the five HIV-2-infected children initiated on ART died and the remainder were lost to follow-up. One of the deaths occurred in a 12-year girl who started ART five months after diagnosis. She had difficulty tolerating the ART with persistent nausea, vomiting and diarrhoea which did not respond to therapy. Her weight dropped to 11.5 kg at 7^th^ week of treatment and she developed sepsis with anaemia and thrombocytopaenia. She died after 2 months on ART. The second death occurred in a 6-year-old boy with unexplained severe anaemia which was refractory to treatment; he died of anaemic heart failure after 4 months on ART.

Among those lost to follow-up, CD4 T-cell recovery was sub-optimal and none achieved virologic suppression. Their clinic visitation was characterised by irregular attendance associated with poor social and treatment support resulting in sub-optimal adherence to therapy. Further details including socio-demographic characteristics of the five HIV-2-infected children are shown in Table 2.

## Discussion

The objective of ART in the treatment of HIV disease is to inhibit viral replication and achieve and sustain viral suppression while promoting immune reconstitution and clinical improvement. There is increasing evidence from sub-Saharan Africa that under routine program conditions, HIV-infected children receiving ART can achieve improved immune function, sustained virological suppression and good clinical outcomes [25]. Ours is the first study to report the early and medium-term immuno-virological outcomes of ART-naïve HIV-1 and HIV-2 infected children starting ART in the concentrated low prevalence setting of The Gambia – West Africa.

Despite a median baseline CD4 T cell percent of 13%, which indicates that the HIV-1 infected children in this cohort were at an advanced stage of immunosuppression at entry, the overall median CD4 T cell percent increased to 23.3% and 27% at six and 12 months respectively, with a ‘plateau’ at 18 and 24 months. By this measure, the MRC paediatric cohort demonstrated an immediate and significant response to therapy. These observed early- and medium-term responses are comparable to reports from a review of 28 paediatric cohorts in Sub-Saharan Africa which showed that CD4% increased within the first year of treatment with a plateau after 12 to 18 months of treatment [25]. When stratified by age however, children < 5 years of age at the start of ART in our cohort achieved a sustained increase in CD4% until 30 months of treatment, after which a decline in the median change was observed. This could be explained partially by the anticipated decrease in CD4% with age during the first 5 years of life [26]. Likewise, children ≥ 5 years at start of ART had an initial increase in absolute CD4 count up to 12 months followed by a more blunted CD4 T cell recovery subsequently. These significant early immunological recovery followed by an apparent mid-term ‘plateau’ in CD4 T cell recovery which we observed in our cohort is comparable to reports in other settings [27,28].

The proportion of HIV-1-infected children who achieved an undetectable viral load, defined as HIV RNA <100 copies/mL, was 63.2% at six months. By 12- and 24- months of therapy, 55.6% and 36.4% of the children in the cohort respectively had undetectable viral loads. However, the proportion increased to 58% at 36 months. Possible explanations for this include the fact that some children who failed therapy were switched on to second-line regimens as stated in the results section, as well as the effect of reinforced adherence counselling. Second-line therapy was however frequently delayed as a result of shortages of appropriate medicines and formulations.

Overall, 62.5% of all deaths in our cohort occurred within the first 90 days, with these early mortality predominantly among those with HIV-1 infection and aged <2 years. This is similar to reports elsewhere in the sub-region where early mortality (i.e. in the first 90 days of ART) is substantially higher compared to later months [29-32]. The estimated 12 months survival probability on ART among the HIV-1-infected children of 89.9% falls within range of 12 month survival probability reported from the review of paediatric cohorts in sub-Sahara Africa mentioned earlier [25]. Our findings show sustained improvement in survival experiences which is independent of the baseline CD4^+^ T cell percentage, therefore reinforcing the obvious benefit of ART in a routine program setting in a resource-limited setting and the need to further scale-up access to ART in children.

We found a very poor outcome among the six HIV-2 infected children after a median of 23 weeks on combination antiretroviral therapy, with three out of six being lost to follow-up and two dying. A review of the available laboratory parameters for treatment monitoring similarly showed poor immunologic and virologic responses in almost all of those children. A couple of factors could have contributed to the poor clinical and immuno-virologic outcomes we observed among the HIV-2 infected children in this report. First, all the children were at an advanced disease stage at diagnosis of their HIV-2 infection, and initiated antiretroviral therapy at a point when they were severely immunocompromised. Given the chronicity of HIV-2 infection, the long duration of infection before these children became eligible for ART at the same CD4 count threshold as HIV-1 infected children might have contributed to the poor immunological recovery. Secondly, despite the progress made in scaling-up ART in West Africa, treatment of HIV-2 infection still remains a challenge and the optimal treatment option for HIV-2 infection is still a subject of several ongoing research studies focussing only on adults. While available data from adult HIV-2 cohorts suggests a sub-optimal response to some of the protease inhibitors (PIs) commonly used in the West African sub-region [33,34], very little is known about HIV-2 therapeutic approach in children. However, from our report, it is difficult to conclude that a sub-optimal response to LPV/r-based regimen that we use might have contributed to the observed poor outcome in view of the small number of cases.

Baseline co-morbidities such as tuberculosis, malnutrition, helminthiasis, anaemia as well as ART toxicity or immune reconstitution inflammatory syndrome (IRIS) particularly in those with advanced immunosuppression at ART initiation are well recognized risk factors for poor outcomes on antiretroviral therapy including early mortality [31,35,36]. Most HIV-infected children in resource-limited settings present at an advanced stage of disease with marked immunosuppression mostly because of widespread lack of laboratory capacity for diagnosis of HIV-infection in children less than 18 months of age, who still harbour maternal HIV-antibodies. Although many of the children in our cohort were children of HIV-infected adults enrolled in a natural history cohort who would have benefitted from early diagnosis, a good number however, were referred from the MRC outpatients’ clinic and hospital ward as well as other health-care facilities due to chronic ill health and at an advanced HIV disease clinic stage.

Adherence to ART is another important determinant of viral suppression and treatment outcome [37], and in children is strongly linked to the availability of treatment support. Although a limitation of this study is that adherence as measured by pill counting and care-giver/self report was not specifically analysed as a variable, the observed immuno-virological responses were objective evidence of the levels of adherence to medications within the cohort. Reddi *et al.*[31] reported that having at least one HIV-positive caregiver provides a protective effect against mortality when compared with care-givers who were untested or HIV-negative especially where this care-giver receives treatment at the same site as such a care-giver. They hypothesize that such a care-giver may be more knowledgeable about symptom management and disease progression and therefore able to provide more informed treatment support. The majority of the children on ART in our cohort had both parents alive and at least one HIV-positive parent; we however did not analyze the effect of care-giver HIV-status on treatment outcomes.

As with most other paediatric HIV treatment programmes in resource-limited settings [38,39], we resorted to using adult, generic fixed-dose combination tablets cut in half or quartered. In the absence of pill-cutters this usually resulted in unequal-sized fractions and could have inadvertently contributed to inappropriate, particularly sub-therapeutic, drug doses particularly with the LPV/r gel capsules [40].

As more countries in the sub-region scale-up access to ART for HIV-infected children, paediatric treatment outcomes data can help guide programme implementation. The level of care provided at our HIV clinic is however not representative of paediatric HIV care in West Africa as patients had the advantage of frequent and regular care provided by highly qualified physicians and counsellors at essentially no cost to the patients, with routine viral load and CD4 cell counts and -percent monitoring. This, coupled with the small size of our paediatric cohort makes it difficult to generalize our findings to other settings in the sub-region. We however experienced similar programmatic challenges such as lack of appropriate paediatric formulations of almost all the antiretroviral drugs especially second-line drugs, and stock-out of at least one drug annually. Over 60% of the children on our cohort were severely immunocompromised at treatment initiation, and the need for multiple pre-ART counselling sessions and prior approval by the national Eligibility committee further contributed to delays in starting treatment which may have put these patients at increased risk of poor immunological recovery, treatment failure and mortality.

## Conclusions

We have observed that the beneficial effects of antiretroviral therapy among Gambian children are sustained in the first 24 months of treatment with a significant improvement in survival experience of up to 36 months. Implementation of diagnostic PCR or an equivalent technology for earlier HIV diagnosis, especially at maternal and child-health clinics, and improved access to paediatric fixed dose formulations to facilitate adherence and appropriate dosing, are key to scaling up access to life-saving ART for HIV-infected children.

## Competing interests

The authors declare that they have no competing interests.

## Authors' contributions

UO coordinated the pediatric HIV clinical activities, performed the statistical analysis and drafted the manuscript. TT contributed to the study design, participated in the statistical analysis and helped to draft the manuscript. JT participated in the statistical analysis. IP contributed to the design of the study. TT, FO and KP participated in the clinical care of the patients. AJ conceived the idea and gave overall support. All authors read and approved the final manuscript.

## Pre-publication history

The pre-publication history for this paper can be accessed here:

http://www.biomedcentral.com/1471-2431/12/95/prepub
